# Supplementary material for: Disability and Participation in Breast and Cervical Cancer Screening: A Systematic Review and Meta-Analysis
Source: Int J Environ Res Public Health. 2022 Aug 2;19(15):9465. doi: 10.3390/ijerph19159465 (PMC9368105; doi:10.3390/ijerph19159465)
Supplement: Supplementary file 1 [file ijerph-19-09465-s001.zip › Supplementary File S1.pdf]

No.	MEDLINE search terms
1.	Disabled persons/or amputees/or hearing impaired persons/or mentally ill persons/ or visually impaired persons/or mentally disabled persons
2.	(disabled person$ or disab$ or disabled people).mp
3.	amputee$.mp
4.	(hearing impaired person$ or hearing impaired people).mp
5.	(mentally disabled person$ or mentally disabled people).mp
6.	(mentally ill person$ or mentally ill people).mp
7.	(visually impaired person$ or visually impaired people).mp
8.	Developmental Disabilities/ or developmental disability$.mp
9.	Mental Retardation/ or mental$ retard$.mp
10.	Mobility Limitation/ or mobility limitation$.mp
11.	Dependent ambulation/ or dependent ambulation.mp
12.	Paraplegia/ or paraplegia.mp
13.	Quadriplegia/ or quadriplegia.mp
14.	Hearing Loss/ or hearing loss.mp
15.	Blindness/ or blindness.mp
16.	Vision Disorders/ or vision disorder$.mp
17.	exp Self-Help Devices/ or assistive technology.mp
18.	Mental Disorders/ or mental disorder$.mp
19.	psychiatric disabilit$.mp
20.	(mental health disabilit$ or mental health impairment$).mp
21.	functional limitation$.mp
22.	activity limitation$.mp
23.	mobility impairment$.mp
24.	vision impairment$.mp
25.	hearing impairment$.mp
26.	cognitive impairment$.mp
27.	intellectual disabilit$.mp
28.	participation limitation$.mp

29. 1 or 2 or 3 or 4 or 5 or 6 or 7 or 8 or 9 or 10 or 11 or 12 or 13 or 14 or 15 or 16 or 17 or 18 or 19 or 20 or 21 or 22 or 23 or 24 or 25 or 26 or 28
30. Mass Screening/
31. Screening.mp.
32. Early detection.mp.
33. Screening programmes.mp
34. screening program*.mp.]
35. detection.mp.
36. programme$.mp.
37. cancer screening.mp.
38. neoplasm screening.mp.
39. cancer detection.mp.
40. screening$.mp.
41. screen*.mp.
42. mammography.mp
43. Mammography/
44. Papanicolaou Test/
45. HPV screening.mp.
46. mammograph*.mp.
47. 30 or 31 or 32 or 33 or 34 or 35 or 36 or 37 or 38 or 39 or 40 or 41 or 42 or 43 or 44 or 45 or 46
48. Breast Neoplasms/
49. Uterine Cervical Neoplasms/
50. 48 or 49
51. 29 and 47 and 50
